# Supplementary material for: Abnormal hyperactivity of specific striatal ensembles encodes distinct dyskinetic behaviors revealed by high-resolution clustering
Source: bioRxiv. 2024 Oct 28:2024.09.06.611664. Preprint. [Version 3] doi: 10.1101/2024.09.06.611664 (PMC11418934; doi:10.1101/2024.09.06.611664)
Supplement: Supplement 1 [file media-1.pdf]

REAGENT or RESOURCE	SOURCE	IDENTIFIER
Chemicals, peptides and recombinant proteins		
6-OHDA hydrochloride	Sigma-Aldrich	H4381
L-ascorbic acid, 99%	Sigma-Aldrich	A92902
3,4-Dihydroxy-L-phenylalanine	Sigma-Aldrich	D9628
Benserazide hydrochloride	Sigma-Aldrich	B7283
Antibodies		
Rabbit anti-TH	Peel Freez Biological	P40101-150
Rabbit anti-GFP Alexa Fluor-488 conjugate	Invitrogen (Molecular Probes)	Cat#A-21311, RRID: AB_221477
Alexa Fluor 594 goat anti-rabbit	Jackson ImmunoResearch Labs	Cat#115-585-045, RRID: AB_2338062
Bacterial and Virus Strains		
AAV5.CAG.Flex.GCaMP6f.WPRE.SV40	University of Pennsylvania Vector Core	Cat#100835-AAV5, #AV-5-PV2816
Experimental Models: Organisms/Strains		
D1-Cre, Tg(Drd1a-cre) FK150Gsat/Mmucd	MMRRC	#029178-UCD
A2a-Cre, B6.FVB(Cg)-Tg(Adora2acre) KG139Gsat/Mmucd	MMRRC	#036158-UCD
Softwares and Algorithms		
Unsupervised behavioral clustering algorithm	This paper; Klaus et al 2017; Frey and Dueck 2007	N/A
CNMF-E	Klaus et al., 2017; Pnevmatikakis et al., 2016; Friedrich et al., 2017; Zhou et al., 2018	N/A
Bonsai 2.4.	Lopes et al., 2015	https://bonsai-rx.org/; RRID:scr_017218
DeepLabCut	Mathis et al., 2018	https://github.com/DeepLabCut/DeepLabCut; RRID: SCR_021391
Python Video Annotator	Champalimaud Foundation	https://github.com/video-annotator/pythonvideoannotator
Spyder 3.2.4	Spyder/Python	https://www.spyder-ide.org; RRID:SCR_017585
GraphPad Prism 9	GraphPad Software	https://www.graphpad.com/; RRID: SCR_002798
Inscopix Data Processing Software	Inscopix Inc.	https://www.inscopix.com/software-analysis-miniscope-imaging
Image J	NIH	https://imagej.nih.gov/ij/index.html; RRID: SCR_003070
MATLAB	MathWorks	https://www.mathworks.com/products.html; RRID: SCR_001622
Adobe Illustrator CS5	Adobe	https://www.adobe.com/products/illustrator.html; RRID: SCR_014198
